# Combined Treatment of *Mori folium* and Mori Cortex Radicis Ameliorate Obesity in Mice via UCP-1 in Brown Adipocytes

**DOI:** 10.3390/nu15173713

**Published:** 2023-08-24

**Authors:** Do-Sung Kim, Hwa-Young Lee, Hwa-Jin Kim, Geum-Hwa Lee, Young Jae Lim, Bo Mi Ko, Ji-Hyun Kim, Tae Won Kim, Hye Kyung Kim, Tae Young Kim, Dae Il Hwang, Ha Kyoung Choi, Seon Min Ju, Myung Ja Chung, Han-Jung Chae

**Affiliations:** 1 Non-Clinical Evaluation Center Biomedical Research Institute, Jeonbuk National University Hospital, Jeonju 54907, Republic of Korea; dosung77@jbnu.ac.kr (D.-S.K.); youngat84@jbnu.ac.kr (H.-Y.L.); tailove2212@naver.com (H.-J.K.); dladudwpp12@gmail.com (Y.J.L.); goboming@naver.com (B.M.K.); kjh96522@naver.com (J.-H.K.); 2 Research Institute of Clinical Medicine, Biomedical Research Institute, Jeonbuk National University, Jeonbuk National University Hospital, Jeonju 54907, Republic of Korea; heloin@jbnu.ac.kr; 3 School of Pharmacy, Jeonbuk National University, Jeonju 54896, Republic of Korea; 4 College of Pharmacy, Kyungsung University, 309 Suyeong-ro, Busan 48434, Republic of Korea; tone127@naver.com (T.W.K.); fiona30@ks.ac.kr (H.K.K.); 5 Institute of Jinan Red Ginseng, Jinan-gun 55442, Republic of Korea; kty54090@ijrg.re.kr (T.Y.K.); dh5366@ijrg.re.kr (D.I.H.); gkrud3327@ijrg.re.kr (H.K.C.); jusm1008@ijrg.re.kr (S.M.J.); 6 Department of Pathology, College of Medicine, Jeonbuk National University, Jeonju 54896, Republic of Korea; mjchung@jbnu.ac.kr

**Keywords:** mulberry leaf, AMPK, obesity, Ucp-1, adipogenesis, browning, functional food

## Abstract

Mori Folium (*Morus alba* leaf, MF) and Mori Cortex Radicis (*Morus alba* root cortex, MR) have been studied for their anti-obesity effects by enhancing the browning process and inhibiting adipogenesis. However, important aspects of their protective mechanisms have not been thoroughly investigated, which could aid in developing functional food. Thus, this study aims to determine the synergistic effects of MF and MR against obesity and its associated mechanisms. In an in vitro cell culture model of brown adipocytes, a 1:1 mixture of MF and MR showed a synergistic effect on the expression of brown adipocyte-specific genes, including *Ucp-1*, *Ppargc1a*, Cbp/p300-interacting transactivator (*Cited*), *Prdm16*, *Tbx1*, and *Fgf21* compared with either MF- or MR-treated conditions. Moreover, they demonstrated the involvement of cAMP and Ca2+ in induction of brown adipocyte-specific genes. In an in vivo model using HFD-fed mice, MF/MR significantly inhibited weight gain, plasma cholesterol, LDL, TG content, fat mass, and adipocyte size. Furthermore, MF/MR inhibited morphological alteration and the expressions of fatty acid synthesis genes such as *Srebp1* and *Fasn* in the white adipose tissue. Thermogenesis genes were recovered in the brown adipose tissue with MF/MR supplementation, indicating that MF/MR regulated adipocytic dysmetabolism where AMPK signaling is involved. In conclusion, these results suggested that MF/MR regulates brown and beige adipocyte processes, providing one of the preventive functional food/herbal medicines against obesity and its associated metabolic diseases.

## 1. Introduction

The rising incidence of obesity is a global concern with profound implications for human health. Primarily caused by factors such as excessive food intake, lack of physical activity, and genetic susceptibility, obesity is linked to various metabolic diseases. These include cardiovascular disease, type 2 diabetes, hyperlipidemia, and hypertension, which not only compromise health but also increase the economic burden on patients. Beige/brown-like adipocytes have a thermogenic function. They express uncoupling protein 1 (UCP-1) in the inner mitochondrial membrane, which can dissipate the heat produced by the proton electrochemical gradient. Subcutaneous white adipocytes (WAT) are more responsive to browning agents than visceral WAT [1]. Therefore, subcutaneous WAT browning has been considered a promising strategy against obesity [2]. Some browning agents, such as rosiglitazone [3], show potent browning activities, but animal investigations have linked rosiglitazone to hepatotoxicity (5 mg/kg, p.o in diabetic mice) and cardiac toxicity (10 mg/kg, in doxorubicin-treated rats) [4]. Additionally, medications prescribed for obesity can have various side effects as they may cause gastrointestinal disturbances such as diarrhea, constipation, nausea, and vomiting. Moreover, the currently accessible medications are not suitable for most individuals, as physicians prescribe them according to the specific health conditions of each individual. The currently available pharmaceutical interventions do not constitute an independent and comprehensive remedy for sustained weight reduction over an extended period. Hence, there is a need for treatment targeting obesity that can be applied to a broader population, offering extended health benefits.

Given these limitations, functional foods derived from natural products known for weight loss effects are gaining popularity due to their safety, low cost, and efficacy. Among these, the mulberry plant stands out. Its leaves, fruits, roots, and root barks are widely used in traditional Chinese medicine and cuisine for their excellent health benefits, particularly against obesity and associated metabolic disorders. Further, there are ecological reasons for mulberry use, including carbon sequestration and soil erosion prevention [5]. There are 11 main species, but the most common species of mulberry are black (*Morus nigra* L.), white (*Morus alba* L.), and red (*Morus rubra* L., also called American mulberry and native to the eastern United States), followed by other species: *Morus australis*, *Morus bomycis*, *Morus laevigata*, *Morus serrata*, *Morus macroura*, *Morus cathayana*, *Morus multicaulis*, and *Morus insignis* [6]. White mulberry has been cultivated and used for various purposes, including as a functional food. *Morus alba* L. (*M. alba*) leaves and roots contain compounds with potential health benefits, such as antioxidants, vitamins, and minerals [7].

*M. alba* is rich in isoflavonoids, but the composition and concentration of isoflavonoids may vary with plant parts. The unique health benefits are influenced not by a single component of the natural product but by the combined effect of all of the components in the natural product. Naturally, all the constituents of natural products produce a biological effect that benefits health [8]. Although *M. alba* has functional components, such as 1-deoxynojirimycin (1-DNJ), morin, moracin, and flavonoids, which contribute to its anti-obesity, antihyperglycemic and anti-inflammatory effects [9], each plant extract has its unique compound composition depending on the main part of the plant used for the extraction process [10]. Therefore, a combination of mulberry plant parts could be extracted separately and combined to have all the main functional components in the extract, which exhibit anti-obesity effects. In this study, the anti-obesity effects of Mori Folium (*Morus alba* leaf, MF) and Mori Cortex Radicis (*Morus alba* root cortex, MR) were evaluated separately. Additionally, the optimal combination of MF and MR at 1:1 was used to evaluate the in vivo effects on obesity in a high-fat diet (HFD) feeding animal model.

## 2. Materials and Methods

### 2.1. Preparation of Mori Folium and Mori Cortex Radicis Extracts

Mori Folium (MF) and Mori Cortex Radicis (MR) were provided by the Institute of Jinan Red Ginseng (Jinan, Republic of Korea). The dried MF (5 kg) was pulverized with a blender and extracted with 50 L of distilled water at 80 °C under reflux for 3 h. Similarly, dried MR (5 kg) was pulverized with a blender and extracted with 50 L of 30% ethanol at 50 °C under reflux for 3 h. Filtered MF and MR were concentrated using a vacuum rotary evaporator (R-124, Rotavapor, Rotary Evaporator, Flawil, Switzerland) and lyophilized with a freeze dryer (FD8512, ilShin BioBase, Ede, The Netherlands). The yield of MF and MR were 20.1% and 18.4%. The lyophilized MF and MR extracts were stored at −80 °C until use. Different solvents were selected for extraction based on preliminary tests and experience. The greater the number of sugar molecules attached to a compound, the greater its water solubility. The presence of sugar molecules in mulberry water led to its selection as one of the solvents. In addition, preliminary experiments demonstrated that 30% ethanol enhanced the extraction efficiency of mulberroside A. Consequently, 30% ethanol was used for this investigation.

### 2.2. HPLC Analysis

Standards and sample solutions were using Hitachi Lachrom Elite^®^ HPLC system (Hitachi High-Technologies Corporation, Tokyo, Japan) equipped with a Zorbax Eclipse C18 analytical column with acetonitrile in solvent (A) and distilled water in solvent (B). The retention time of rutin (Sigma-Aldrich, St. Louis, MO, USA), isoquercitrin (Sigma-Aldrich, St. Louis, MO, USA), astragalin (Sigma-Aldrich, St. Louis, MO, USA), mulberroside A (Sigma-Aldrich, St. Louis, MO, USA), and *cis*-mulberroside A (Sigma-Aldrich, St. Louis, MO, USA) were compared with those in MF and MR and their mixtures (MF/MR). Acetonitrile was used in solvent A and distilled water was used in solvent B. HPLC conditions used for the analysis of standards are shown in Supplementary Table S1. All the standards used in the study were obtained from Sigma Aldrich, St. Louis, MO, USA.

### 2.3. Reagents and Antibodies

Antibodies against p-AMPK (#P-2535) and AMPK (#2532) were purchased from Cell Signaling Technology (Danvers, MA, USA). Antibodies against UCP-1 (#Cat sc-293418), PGC-1α (#Cat sc-518038), p-CREB (#Cat sc-81486), CREB (#Cat sc-271), PPAR-γ (#Cat sc-271392), C/EBPα (#Cat sc-166258), p-HSL (#Cat sc-139656), HSL (#Cat sc-74489), FAS (#Cat sc-74540), SIRT-1 (#Cat sc-74540), SREBP-1c (#Cat sc-36553), and β-actin (#Cat sc-47778) were purchased from Santa Cruz Biotechnology (Santa Cruz, CA, USA). Compound C (CC), H-89, and BAPTA-AM were purchased from Sigma-Aldrich (St. Louis, MO, USA).

### 2.4. 3T3-L1 Cell Culture, Differentiation, and Browning Process

**Figure 1:**
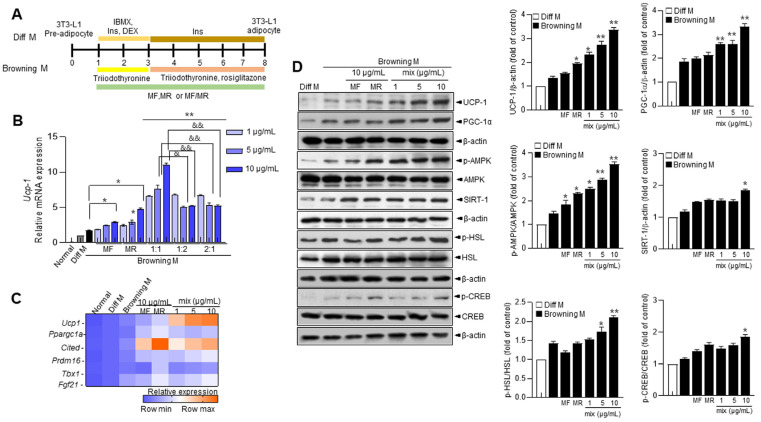
Browning induction and effects of MF/MR on thermogenesis-associated mRNA and protein expressions in 3T3-L1 adipocytes. 3T3-L1 preadipocytes were incubated with normal medium and differentiation medium for 7 days and maintenance and browning medium for 2 days and the subsequent 5 days, and then treated with 1, 5, and 10 μg/mL MF and MR or MF/MR at the ratio, 1:1, 2:1, and 1:2. (**A**) Schematic representation of browning induction and specific treatment at different time intervals. (**B**) *Ucp1* mRNA expression was measured by real-time RT-PCR. (**C**) Heat map clustering analysis of brown fat cell-specific genes such as *Ucp1*, *Ppargc1a*, *Cited*, *Prdm16*, *Tbx1*, and *Fgf21*. (**D**) Immunoblotting of UCP-1, PGC1α, p-AMPK, AMPK, and SIRT1, and their quantification. Data are presented as mean ± SEM (* *p* < 0.05; ** *p* < 0.001 compared to browning medium-treated group; & *p* < 0.05; && *p* < 0.001 compared to each counter concentration of 1:1 MF/MR group). Normal—normal medium group; Diff M—differentiated medium group; Browning M—browning medium-treated group. MF—Mori Folium extract, MR—Mori Cortex Radicis extract, Mix—1:1 mixture of MF/MR.

3T3-L1 preadipocytes were purchased from the ATCC (Manassas, VA, USA) and were cultured in DMEM (Gibco, Carlsbad, CA, USA), containing 1% penicillin/streptomycin, and 10% newborn calf serum (NCS, Gibco, Carlsbad, CA, USA), and cultured in a humidified incubator maintained at 37 °C with 5% CO_2_. 3T3-L1 preadipocytes were grown to 100% confluence under standard culture conditions (day 1). The cells were treated with differential medium containing 10% fetal bovine serum (FBS, Gibco, Carlsbad, CA, USA), 5 µg/mL insulin, and maintained in 10% FBS DMEM containing insulin for another 5 days (day 3–8). For induction of browning in 3T3-L1 adipocytes, they were treated with a medium containing 50 nM triiodothyronine (T3, sigma) (day 1–3), and maturation medium was supplemented with 50 nM triiodothyronine and 1 μM rosiglitazone (Rosi, Abcam, Cambridge, UK) (day 3–8), called browning media [11]. ML and MR were dissolved in the culture medium to prepare a stock solution. To induce browning, cells were treated with 1, 5, and 10 µg/mL ML, MR, or the combination of ML and MR extract at 1:1, 1:2, and 2:1 (described in Figure 1A).

### 2.5. Cell Viability under Different Conditions

3-(4,5-dimethylthiazol-2-yl)-2,5-diphenyltetrazolium bromide (MTT, Sigma, St. Louis, MO, USA) assay was used to find out the effect of MF and MR on cell viability. Briefly, cells were treated with MF or MR (0–100 μg/mL) for 24, 48, and 72 h in 96-well plates. Then, 100 μL MTT solution (5 mg/mL) was added to each well containing culture media and incubated for 4 h at 37 °C. Cells were then exposed to dimethyl sulfoxide (DMSO), and the absorbance was recorded at 540 nm using a microplate reader (Thermo Scientific, Waltham, MA, USA). The test was repeated three times.

### 2.6. Animal Experiments

The animal investigations involved in the study were approved by the Institutional Animal Care and Use Committee (IACUC) of Jeonbuk National University Hospital (JBUH-IACUC-2021-14); 7-week old male C57BL/6 mice were procured from Orient Science Co. (Seongnamsi, Gyeonggi-do, Republic of Korea). Mice were housed and cared for by the following laboratory animal care guidelines [12]. The mice were allowed for a week to adapt to local standard conditions on a standard chow diet. After acclimatization, the animals were divided into five groups, each with 8 animals. The study groups are as follows: NCD control group, mice fed with vehicle (water) and normal chow diet (NCD) containing 15.8% fat; MF/MR group, NCD with 200 mg/kg MF/MR; MF/MR100, mice fed with vehicle (water) and high-fat diet (HFD) containing 1% cholesterol, 18% lipid, 40% sucrose, 1% AIN-93G vitamins, and 19% casein, and HFD mice supplemented with 100 mg/kg of MF/MR; MF/MR200, HFD mice supplemented with 200 mg/kg of MF/MR. All the mice were fed with NCD or HFD with vehicle or 100 or 200 mg/kg MF/MR once daily by oral gavage for 9 weeks. During the intervention period, food intake and body weight were recorded once per week. At the 8th week, the mice were anesthetized, and their fat mass was analyzed with dual-energy X-ray absorptiometry (DXA, iNSiGHT VET DXA, OsteoSys Crop., Guro-Gu, Seoul, Republic of Korea). Thermal images were obtained using the FLIR ONE camera (FLIR Systems, Inc., Wilsonville, OR, USA) attached to an iPhone (model 11, Apple, Inc., Los Altos, CA, USA). After 9 weeks, the mice were fasted overnight prior to euthanasia with an intraperitoneal injection of 100 mg/kg ketamine. Immediately after euthanasia, whole blood, white adipose tissue (abdominal visceral and epididymal), interscapular brown adipose tissue, and liver were collected. The collected whole blood was centrifuged to collect serum. All other samples were stored at −80 °C for further analysis.

### 2.7. Biochemical Analysis

Serum insulin concentration was measured by using IDDM2 ELISA kit (CUSABIO, Houston, TX, USA). Alanine aminotransferase (ALT), aspartate aminotransferase (AST), creatinine, glucose, and lipids, including triglyceride (TG), total cholesterol (TC), and LDL-cholesterol (LDL-C) in the tissue, were analyzed using automated biochemical analyzer (Asan Pharmaceutical, Seoul, Republic of Korea). The test was repeated three times.

### 2.8. Histological and Immunohistochemical Analysis

Histological and immunohistochemical analyses were performed as previously described [13]. Briefly, adipose tissue samples were fixed with 10% formalin, embedded in paraffin, and sectioned at 4 µm. Then, sections were stained with hematoxylin and eosin (H&E). All the immunohistochemical detections were processed as described previously [14]. All the acquired images were examined with Image J 1.41 software (National Institutes of Health, Bethesda, MD, USA).

### 2.9. RNA Isolation and Quantitative Real-Time RT-PCR

Total RNA from cells and tissue were isolated with TRIzol (Invitrogen, Carlsbad, CA, USA). Then, reverse transcription was performed with 2 µg of RNA with oligo dT primers. qRT-PCR was conducted with Power SYBR™ Green PCR Master Mix (Applied Biosystems, Foster City, CA, USA). Quantification was performed by a comparative cycle threshold (Ct) method, and the resultant product was corrected for the amount of *β-actin* expression. The primers used in this study are listed in Supplementary Table S2. The test was repeated three times.

### 2.10. Immunoblotting

Immunoblotting was performed as reported earlier [15]. The tissue homogenates and cell lysates were determined using the Bradford (Bio-Rad, Hercules, CA, USA) method and equal amounts of protein (20 μg) separated with 8–12% SDS-PAGE and transferred to a PVDF membrane via a semi-dry transfer system from Bio-Rad (Hercules, CA, USA). The membranes were blocked with 5% skim milk and incubated overnight with the antibody as directed. Protein signals were developed with ECL kit (Bio-Rad, Hercules, CA, USA).

### 2.11. Dual-Energy X-ray Absorptiometry (DXA) Scan

DXA scan was performed as described earlier [16]. The percentages of fat and fat mass were estimated using a cone-beam flat panel detector DXA (iNSiGHT VET DXA, OsteoSys Crop., Guro-Gu, Seoul, Republic of Korea) as per the guidelines suggested by the manufacturer. The whole-body scan was carried out to identify the region of interest (ROI) for assessing abdominal obesity. The proportion of abdominal fat was calculated using the formula, abdominal fat (DEXA)/total fat (DEXA) × 100.

### 2.12. Immunohistochemistry

The tissue sections were deparaffinized, xylene removed with ethanol, hydrated in graded alcohols, and placed in deionized water for 5 min. For immunostaining, antigen retrieval was conducted using 0.05% trypsin solution for 20 min at 37 °C, and the sections incubated in 3% hydroperoxide for 10 min and incubated overnight with primary antibodies against UCP-1 (Santa Cruz, Dallas, TX, USA) at 4 °C. After washing, sections were incubated with the horseradish peroxidase (HRP)-conjugated secondary antibody. The HRP-conjugated antibody was visualized with a 3,3’-diaminobenzidine tetrahydrochloride (DAB) kit (DAKO, Carpinteria, CA, USA). The sections were counterstained with hematoxylin and imaged using an optical EVOS M5000 imaging system (Invitrogen, Waltham, MA, USA).

### 2.13. Statistical Analysis

The statistical analyses were performed using GraphPad Prism version 8.0 (GraphPad Software, San Diego, CA, USA). The data were determined using one-way analysis of variance (ANOVA) and followed by Tukey’s multiple comparisons test, where *p* < 0.05 is considered significant. Data are shown as mean ± SEM.

## 3. Results

### 3.1. Mori Folium (MF) and Mori Cortex Radics (MR) Extracts Promote the Expressions of UCP-1 and Browning-Associated Genes and Proteins in 3T3-L1 Cells

To determine the non-toxic concentrations of MF and MR on 3T3-L1 cells, we treated the cells with various concentrations of MF and MR (0–100 μg/mL) for 24, 48, and 72 h. Supplementary Figure S1A shows that cell viability remained unaltered at all described concentrations of MF- and MR-treated cells at 24 h but significantly decreased at relatively high concentrations (>10 μg/mL) after 48 h and 72 h. However, the viability remained above the level of IC50, even at the highest concentration, 100 μg/mL. When we combined MF/MR at the ratios 1:1, 1:2, and 2:1, concentrations < 25 μg/mL showed no toxicity to cells, while concentrations > 25 μg/mL appeared to be toxic, with toxicity increasing with concentration (Figure S1B). Therefore, we used the non-toxic concentration of 10 μg/mL MF and MR throughout the in vitro studies.

To confirm the role of MF, MR, and their mixture, we differentiated 3T3-L1 preadipocytes under differentiated media (Diff. M) and browning cocktail agents such as 50 nM triiodothyronine and 1 µM rosiglitazone, referred to as browning medium (Browning M). We then treated the cells with MF, MR, or MF/MR at the ratios of 1:1, 1:2, and 1:3, followed by browning medium, shown by a scheme (Figure 1A). Thermogenesis-related genes, *Ucp1* and *Ppargc1a* (its matched human gene name; PPAR-γ co-activator-1α (PGC-1α), exhibited high expression under the browning condition (Figure S2A,B). Interestingly, the *Ucp1* and *Ppargc1a* expressions significantly increased in the cells treated with MF/MR at the ratios of 1:1, 1:2, and 2:1 compared to the DM-cock-maintained condition (Figure 1B,C). In particular, Ucp1 was more significantly expressed in the MF/MR mixture at the 1:1 ratio than at the 1:2 and 2:1 ratios.

Considering that increased energy consumption and regulation of thermogenesis in mitochondria-rich brown adipose tissue (BAT) occur upon Ucp1 expression [17], we performed heat map clustering analysis for brown fat-specific genes, including *Ucp1*, *Ppargc1a*, *Cited*, PR domain containing 16 (*Prdm16*), T-box transcription factor (*Tbx1*), and Fibroblast growth factor 21 (*Fgf21*). The MF/MR mixture treatment showed a synergistic effect relative to the maximum concentration-treated MF or MR in a concentration-dependent manner (Figure 1C). Furthermore, MF/MR significantly increased protein expression of UCP-1 and PGC-1α, as well as p-AMPK, SIRT-1, p-HSL, and p-CREB, compared with MF or MR (Figure 1D). This provided evidence for the effect of MF/MR on BAT mitochondria metabolism-associated proteins, as well as gene expressions [18].

### 3.2. Supplementation of MF/MR Stimulates the Expression of UCP-1 through AMPK Signaling, Including Ca^2+^ and cAMP

**Figure 2:**
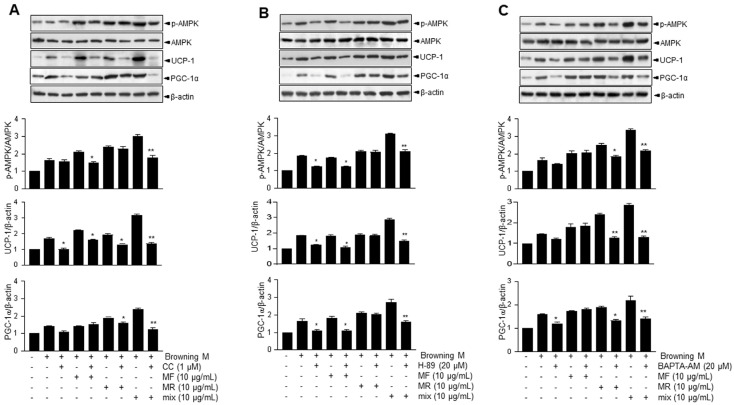
Effects of Mole Folium and Mori Cortex Radicis extracts on the AMPK pathway through PKA and calcium in 3T3-L1 cells. 3T3-L1 preadipocytes were incubated with normal medium, differentiation medium, and browning medium for 7 days and then treated with 1, 5, and 10 μg/mL MF and MR or MF/MR at the ratio, 1:1 with or without 1 μM compound C, 20 μM H-89, and 20 μM BAPTA-AM. (**A**) Immunoblotting of p-AMPK, AMPK, UCP-1. PGC-1α and β-actin and respective quantification (lower panel) in cells treated with compound C. (**B**) Immunoblotting of p-AMPK, AMPK, UCP-1. PGC-1α and β-actin and respective quantification (lower panel) in cells treated with H-89. (**C**) Immunoblotting of p-AMPK, AMPK, UCP-1. PGC-1α and β-actin and respective quantification (lower panel) in cells treated with BAPTA-AM. Data are presented as mean ± SEM (* *p* < 0.05, ** *p* < 0.001, compared to browning medium-incubated cells in the absence of each inhibitor). Diff M—differentiated medium group; Browning M—browning media-treated group; MF—Mori Folium extract; Mix—1:1 mixture of MF/MR; CC—compound C.

UCP-1 and PGC-1α are known downstream effectors of AMPK-dependent metabolic signaling [19]. Thus, AMPK phosphorylation was measured. To determine whether AMPK activation is associated with thermogenesis metabolism in MF/MR-treated cells, we pretreated 3T3-L1 cells with compound C (CC), a recognized AMPK inhibitor, before administering MF, MR, or MF/MR. As depicted in Figure 2A, MF/MR significantly increased the levels of p-AMPK and subsequent thermogenic proteins, including UCP1 and PGC1α, compared to MF or MR alone. These effects were nearly eliminated in the presence of CC, reducing them to control levels.

To gain more insight into the activation mechanism of AMPK, in which cyclic AMP-linked PKA and Ca^2+^-based calcium/calmodulin-dependent protein kinase (CaMKK) are involved [20], we applied H-89, a known PKA inhibitor, to cells treated with MF, MR, or MF/MR. The ratio of p-AMPK/AMPK and the expressions of UCP1 and PGC1, which were increased by MF/MR, were significantly reduced in the presence of H-89 (Figure 2B). The MF-induced increase in p-AMPK, UCP1, and PGC1α expressions were significantly reduced under PKA inhibition. Interestingly, H89 did not influence the expressions of p-AMPK and PGC1α in the presence of MR, suggesting that the PKA involvement is primarily due to MF, not MR (Figure 2B).

Next, we assessed whether BAPTA-AM, a known cytosolic calcium chelator, would attenuate the effects of MF/MR on the expressions of p-AMPK, UCP1, and PGC1α. As expected, BAPTA-AM suppressed the MF/MR-induced activation of AMPK and its linked expressions of UCP1 and PGC1α (Figure 2C). Similarly, MR-induced increases in p-AMPK, UCP1, and PGC1α expressions were also significantly reduced under Ca^2+^ chelation. However, BAPTA-AM treatment did not influence the protein expressions in the presence of MF, indicating that the Ca^2+^ involvement is largely attributable to MR, not MF.

### 3.3. Analysis of Compounds in MF, MR, and MF/MR

We tested analytical interference by comparing standards with MF, MR, and their mixture (MF/MR). HPLC analysis revealed the patterns of rutin, isoquercitrin, and astragalin in MF and MF/MR (Supplementary Figure S3A,B). Furthermore, mulberroside A and cis-mulberroside A in MR and MF/MR were well separated without being disrupted by other peaks. To prove the linearity of the method, we calculated the calibration curves of standards as regression equations (*y = ax + b*). The correlation coefficients (R2) of all compounds’ standards were found to be greater than 0.99 (Supplementary Table S3). Using these demonstrated linear calibration curves, rutin, isoquercitrin, astragalin, mulberroside A, and cis-mulberroside A were quantified. The concentrations of rutin, isoquercitrin, and astragalin in MF were found to be relatively low, although their concentrations were approximately twice as high as those observed in MF/MR. Interestingly, in MR and MF/MR, the concentration of cis-mulberroside A was higher than that of mulberroside A. Moreover, the concentrations of mulberroside A and cis-mulberroside A in MR were approximately twice those observed in MF/MR (Table 1).

### 3.4. Supplementation of MF/MR Regulate Body and Adipose Tissue Weights, and Lipid Metabolism in HFD-Induced Obese Mice

**Figure 3:**
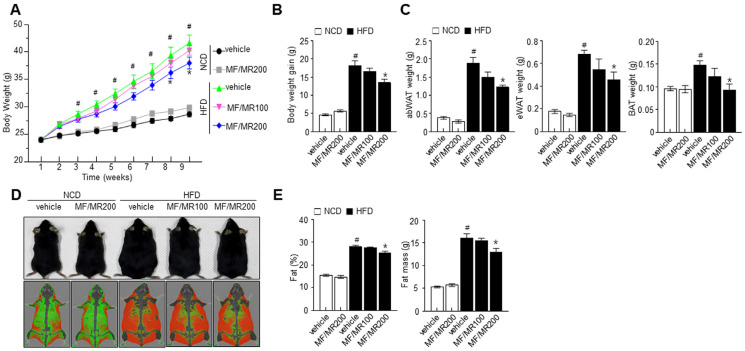
Synergistic effects of MF/MR on body weight and fat mass in HFD-fed mice. Mice fed with normal diet were administered with vehicle and 200 mg/kg MF/MR, whereas HFD-induced obese mice were treated with vehicle and 100 and 200 mg/kg MF/MR. All the animals were fed daily for 9 weeks. Weekly body weight during the experimental period (9 weeks) (**A**) and body weight gain at the end of the experiment (**B**) were measured. (**C**) abWAT, eWAT, and BAT weights were measured. (**D**) Representative DXA scan images showing fat distribution and (**E**) calculated fat percentage and mass. Data are presented as mean ± SEM. # *p* < 0.05 indicates a significant difference with NCD and * *p* < 0.05 indicates a significant difference with HFD. NCD—normal chow diet; HFD—high-fat diet; MF/MR—1:1 mixture of MF/MR; Vehicle—normal group.

**Figure 4:**
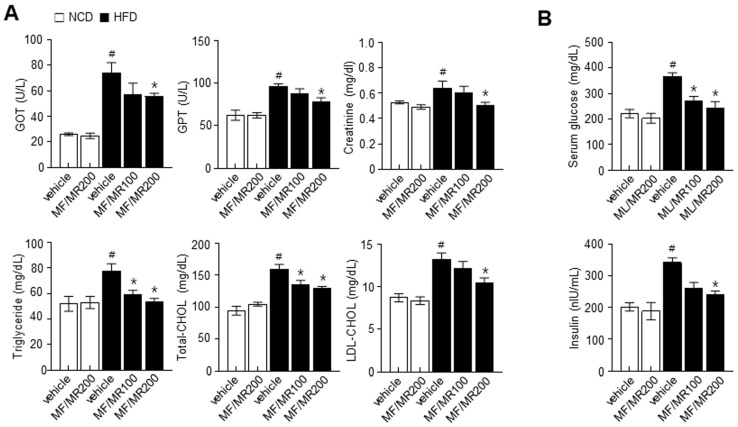
Synergistic effects of MF/MR on serum biochemical profiles in HFD-fed mice. Mice fed with normal diet were administered with vehicle and 200 mg/kg MF/MR, whereas HFD-induced obese mice were treated with vehicle and 100 and 200 mg/kg MF/MR. All the animals were fed daily for 9 weeks. (**A**) Levels of GOT, GPT, creatinine, triglyceride, total cholesterol, and LDL-cholesterol. (**B**) Measurement of fasting glucose and insulin at the end of the experiment. Data are presented as mean ± SEM (# *p* < 0.05, compared to NCD, and * *p* < 0.05, compared to HFD group). NCD—normal chow diet; HFD—high-fat diet; MF/MR—1:1 mixture of MF/MR; Vehicle—normal group; GOT—glutamic oxaloacetic transaminase; GPT—glutamic-pyruvic transaminase; LDL—low-density lipoprotein.

To investigate the effect of MF/MR on obesity, we administered MF/MR to HFD-induced obese mice for nine weeks. After the dietary intervention, HFD-induced obese mice that had not received MF/MR showed a significantly higher weight gain than the NCD mice. However, obese mice supplemented with MF/MR had a lower weight than their counterparts without MF/MR supplementation (Figure 3A,B). In addition, supplementation with MF/MR attenuated the weight of abdominal, epididymal WAT, and interscapular BAT (Figure 3C). A DXA scan suggested a reduction in fat in obese mice supplemented with MF/MR (Figure 3D,E). Biochemical analysis showed a significant reduction in GOT, GST, creatinine, TG, TC, and LDL-C levels in HFD mice supplemented with MF/MR, as compared to vehicle-treated HFD mice (Figure 4A). To ascertain if MF/MR affects insulin sensitivity, we measured fasting glucose and serum insulin levels. The fasting glucose and insulin levels were significantly lower in HFD mice administered with 200 mg/kg MF/MR than in vehicle-treated HFD mice (Figure 4B).

### 3.5. Administration of MF/MR Reduce Lipid Accumulation and Increase Thermogenesis in HFD-Induced Obese Mice

**Figure 5:**
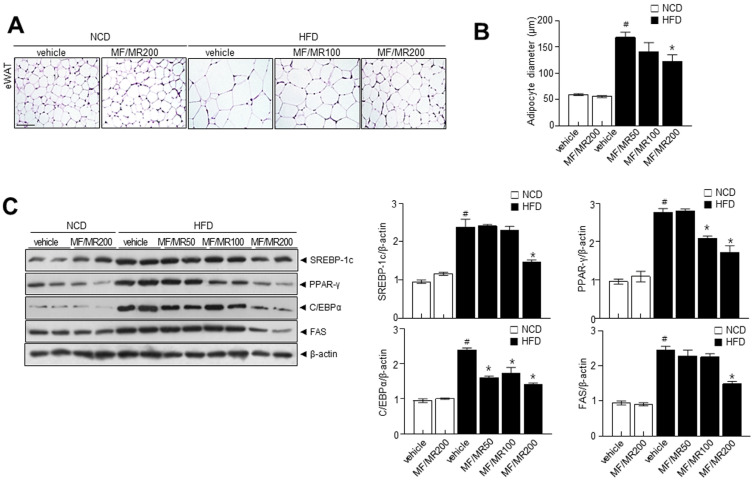
Combined effects MF/MR on eWAT, adipocyte diameter, and adipogenesis protein expressions in HFD-fed mice. Mice fed with normal diet were administered with vehicle and 200 mg/kg MF/MR, whereas HFD-induced obese mice were treated with vehicle and 100 and 200 mg/kg MF/MR. All the animals were fed daily for 9 weeks. (**A**) Representative images stained with H&E. (**B**) The average diameter of adipocytes in eWAT. (**C**) Immunoblotting of SREBP-1c, PPARγ, FAS, and C/EBPα in eWAT, and respective quantification (right). Data are presented as mean ± SEM (# *p* < 0.05, compared to NCD, and * *p* < 0.05, compared to HFD group). NCD—normal chow diet; HFD— high-fat diet; MF/MR—1:1 mixture of MF/MR; Vehicle—normal group; WAT—white adipose tissue. Scale bar: 100 μm.

**Figure 6:**
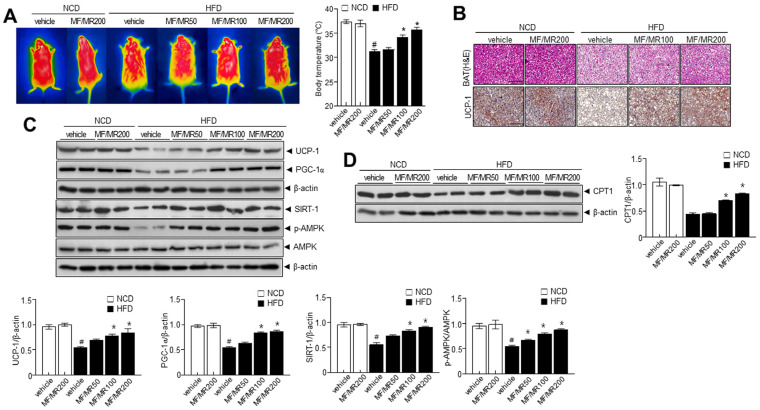
Protective effects of MF/MR in BAT of HFD-fed mice. Mice fed with normal diet were administered with vehicle and 200 mg/kg MF/MR, whereas HFD-induced obese mice were treated with vehicle and 100 and 200 mg/kg MF/MR. All the animals were fed daily for 9 weeks. (**A**) Representative thermal images showing the body temperature of HFD-fed mice (right). (**B**) Representative images stained with H&E and separately immunostained with anti-UCP1 antibody. (**C**) Immunoblotting was performed in BAT using antibodies against SIRT1, p-AMPK, AMPK, UCP-1, PGC-1α, and β-actin and respective quantification (lower panel). (**D**) Immunoblotting of CPT1 and β-actin and respective quantification (right panel). Data are presented as mean ± SEM (# *p* < 0.05, compared to NCD, and * *p* < 0.05, compared to HFD group). BAT—brown adipose tissue; NCD—normal chow diet; HFD—high-fat diet; MF/MR—1:1 mixture of MF/MR; Vehicle—normal group; CPT1—carnitine palmitoyl transferase I.

In eWAT, the diameter of adipocytes in HFD mice supplemented with MF/MR was lower than in HFD mice (Figure 5A,B). We then measured the expressions of adipogenic transcription factors to assess the potential anti-obesity effect of MF/MR in eWAT. The administration of MF/MR markedly decreased the adipogenesis protein expressions of SREBP-1c, PPAR-γ, C/EBPα, and FAS in eWAT from HFD-induced obesity mice (Figure 5C). Additionally, we performed a cold tolerance test to evaluate adaptive thermogenesis in the MF/MR supplemented HFD mice. Acute cold exposure resulted in a significant up-regulation of body temperature in all the groups. However, MF/MR supplementation significantly increased the body temperature compared to the HFD group after 12 h of exposure to 4 °C (Figure 6A). Immunohistochemical analyses revealed that UCP-1 expression was higher in the MF/MR supplemented group compared to the HFD group (Figure 6B). Also, administration of MF/MR significantly increased protein expression of UCP-1 and PGC-1α (Figure 6C). In the iBAT, supplementation with MF/MR restored the decreased ratio of p-AMPK/AMPK and expression of SIRT-1. Further, carnitine palmitoyltransferase 1 (CPT1) expression decreased in HFD mice, but it was restored in the HFD mice that had been administered MF/MR (Figure 6D).

## 4. Discussion

Functional foods derived from natural products that promote health are safe, affordable, and have long-term health benefits. However, the selection of a natural product to treat a specific disorder is crucial. This study demonstrated that extracts of mulberry leaf, roots, and their mixture potentially protect against obesity by elevating thermogenesis. Additionally, the study showed that combining leaf and root extract was the most effective against obesity through adipocyte browning and thermogenesis. Specifically, MF/MR at a ratio of 1:1 significantly increased expressions of thermogenesis genes, and proteins, with Ca^2+^ and PKA, are suggested to contribute to the AMPK and UCP-1 signaling pathway, thereby facilitating adipocyte browning. These findings suggest that MF/MR modulates brown/beige adipocyte processes, potentially making it one of the functional foods that can regulate obesity and its associated metabolic disorders.

Previously, mulberry leaf and root extracts were suggested to reduce obesity and other metabolic disorders such as dyslipidemia and hyperglycemia [21], fibrosis, oxidative stress, and inflammation [22], but its protective mechanism has not been thoroughly studied. Here, MF/MR treatment significantly increased *Ucp1* expression and its related signaling, reflecting its importance in the browning process in 3T3-L1 adipocytes (Figure 1A). In a concentration-dependent manner, *Ucp1* and its related thermogenesis genes were significantly regulated in MF/MR conditions (Figure 1B). Moreover, observations demonstrate that MF/MR treatment positively influenced the AMPK-SIRT-1-UCP1 axis, where UCP1 appears to be the center of BAT thermogenesis and essential to systemic energy homeostasis (Figure 1C). Multiple reports indicate that mulberry and its flavonoid stimulated AMPK in adipocytes and muscle cells, supporting these observations [23]. The AMPK is a well-recognized energy sensor which plays an important role in regulating cellular energy homeostasis [24]. AMPK activation promoted thermogenesis in both brown and WAT [25], whereas AMPK ablation resulted in cold intolerance and a reduction in non-shivering thermogenesis in mouse adipocytes [26]. Importantly, compound C, a commonly used chemical inhibitor of AMPK, effectively suppressed the MF/MR-stimulated SIRT1-UCP-1 axis, indicating that AMPK is required for the induction of browning in WAT (Figure 2A). In this study, PKA and Ca^2+^ involvements seem to be attributable to the AMPK-SIRT1-UCP1 axis in adipocytes. In line with our hypothesis, cAMP/PKA and Ca^2+^ pathways play a central role in increasing adipose tissue browning and thermogenesis [27]. MF/MR remarkably increased AMPK-SIRT1-UCP1 activation, which was controlled by H-89, a PKA inhibitor, and BAPTA-AM, Ca^2+^ chelator (Figure 2B,C), revealing that the regulation of the browning process by MF/MR was closely related to the cytosolic Ca^2+^ and PKA. Moreover, in line with the synergism concept, two compounds can function independently and exhibit their effects collectively. Here, the mechanism involving PKA is linked to MF, whereas Ca^2+^ signaling is linked to MR, and together, they positively impact UCP expression and the browning process. In the study, the browning protocol increased the expression of *Ucp1* and other genes associated with thermogenesis and reduced the overall fat mass of HFD mice treated with MF and MR extracts. These findings suggest that using MF, MR or MF and MR in the browning protocol could represent a novel, natural, and effective strategy against obesity.

Consistently, in vivo studies strongly support the in vitro investigations. MF/MR supplementation in HFD-induced obese mice significantly decreased their weight gain, total adipose tissue weight, and fat mass compared to HFD mice. In this investigation, MF/MR supplementation exhibited down-regulation of SREBP-1C, PPAR-γ, C/EBPα, and its downstream FAS in eWAT than the HFD group (Figure 5C). These findings support the notion that MF/MR supplementation reduces eWAT adipogenesis in HFD-induced obese mice. Several transcriptional factors, PPAR-γ, C/EBPα, and SREBP-1c, mediate adipogenesis [28], influencing the subsequent proteins, such as FAS and ACC [29]. Further, observations suggest that the browning process was enhanced through the AMPK-SIRT1-UCP1 in the MF/MR group (Figure 6C). Similarly, the T2DM (type 2 diabetes mellitus) rats supplemented with mulberry leaf demonstrated brown adipocytes and elevated expressions of UCP1 protein in BAT [30].

Also, mulberry leaf plays a significant role in activating BAT and inducing the browning of WAT in vivo, where the expression of several brown adipocyte marker genes, including *Ucp1*, *Ppargc1a*, *Ppara*, *Prdm16*, and *Cited*, were significantly up-regulated in both iWAT and BAT after mulberry leaf treatment. Likewise, ethanol extracts of mulberry root bark showed potent inhibitory activities on transcription factors and metabolic enzymes, including PPARγ, C/EBPα, SREBP-1c, FAS, and ACC in adipogenesis and lipogenesis. In in vitro study, UCP-1 expression by MF/MR was identified as a promising candidate for a major thermogenic factor. The increased pattern of UCP-1 and its thermogenesis protein expressions were significantly recovered in iBAT of mice supplemented with MF/MR (Figure 6B,C). UCP-1 activation increases body temperature and energy consumption in HFD-induced obese mice [31]. Here, MF/MR supplementation triggered an increase in body temperature and energy expenditure as a result of UCP-1 activation. Throughout this in vivo study, unique MF/MR components have been involved in the anti-obesity function with a controlling effect against adipogenesis and browning. 

The current investigation has a few limitations. First, Ucp1 knock-out (KO) mice were not utilized to understand the mechanism completely. Second, no correlation study was conducted between MF/MR and endogenous components for in vitro and in vivo studies. In the near future, conducting a thorough investigation on the combined effect of MF and MR in knock-out mice is essential.

## 5. Conclusions

In this study, different parts of the mulberry plant were used to produce extracts with various functional components, which were combined to exert a protective effect against obesity. Collectively, the study results suggest that a mixture of 1:1 MF and MR forms one of the functional foods that can prevent obesity and its associated metabolic diseases. Therefore, clinical trials are essential in the near future.

## Figures and Tables

**Table 1:** Standard functional components in MF, MR, and MF/MR.

Sample	Contents of Standards (%)				
	Rutin	Isoquercitrin	Astragalin	Mulberroside A	*Cis*-Mulberroside A
MF	0.003	0.005	0.007		
MR				10.99	13.15
MF/MR	0.002	0.003	0.004	6.68	8.24

MF—Mori Folium extract; MR—Mori Cortex Radicis extract; MF/MR—1:1 mixture of MF/MR.

## Data Availability

The data presented in this study are available upon request from the corresponding author.

## Supplementary Materials

The following supporting information can be downloaded at: https://www.mdpi.com/article/10.3390/nu15173713/s1, Figure S1. Effects of Mori Folium (MF) and Mori Cortex Radicis (MR) extracts on 3T3-L1 adipocytes viability; Figure S2. Thermogenesis gene expressions at the differentiation and browning medium-treated 3T3-L1 adipocytes; Figure S3. Characteristic analysis of bioactive components in extracts of Mori Folium leaf and Mori Radicis Cortex roots by high-performance liquid chromatography; Table S1. HPLC condition for analysis of standards; Table S2. Real time PCR primer sequences; Table S3. Calibration standards for bioactive compounds.
