# Supplementary material for: Combined Treatment of Mori folium and Mori Cortex Radicis Ameliorate Obesity in Mice via UCP-1 in Brown Adipocytes
Source: Nutrients. 2023 Aug 24;15(17):3713. doi: 10.3390/nu15173713 (PMC10489681; doi:10.3390/nu15173713)
Supplement: Supplementary file 1 [file nutrients-15-03713-s001.zip › nutrients-2491990-supplementary.pdf]

Supplementary Figures and Tables

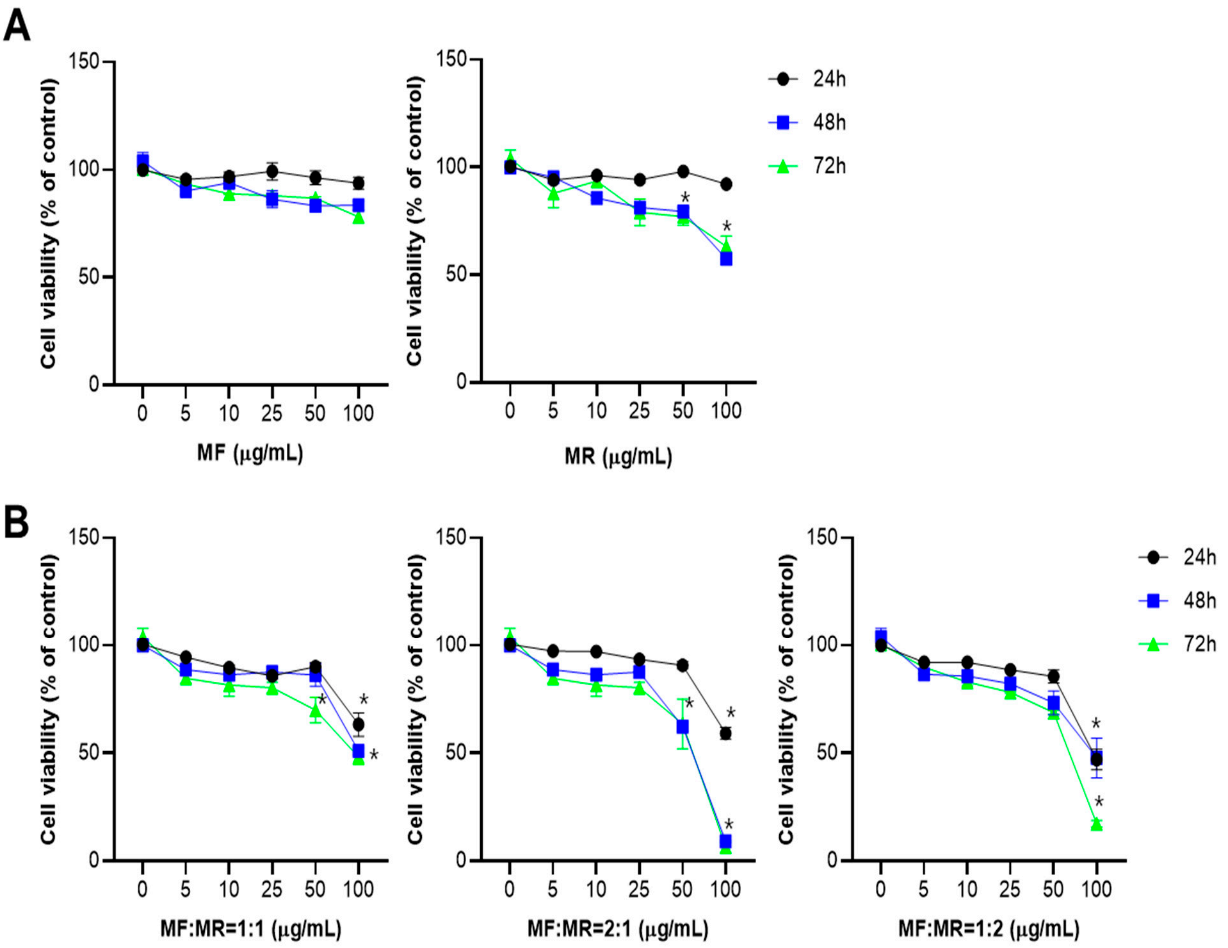

Supplementary Figure S1. Effects of Mori Folium (MF) and Mori Cortex Radicis (MR) extracts on 3T3-L1 adipocytes viability. 3T3-L1 preadipocytes were treated with 0, 5, 10, 25, 50 and 100 μg/ml of ML and MR or ML/MR extract at the ratio, 1:1, 2:1 and 1:2 during 24, 48, and 72 h. (A-B) Cell viability was measured in different conditions. MTT assay was performed. Data are presented as mean ± SEM (*, $p < 0.05$, compared to 0 h at each indicated on centration-treated group, respectively). ML; Mori Folium extract, MR; Mori Cortex Radicis extract, MF/MR; 1:1 mixture of MF/MR.

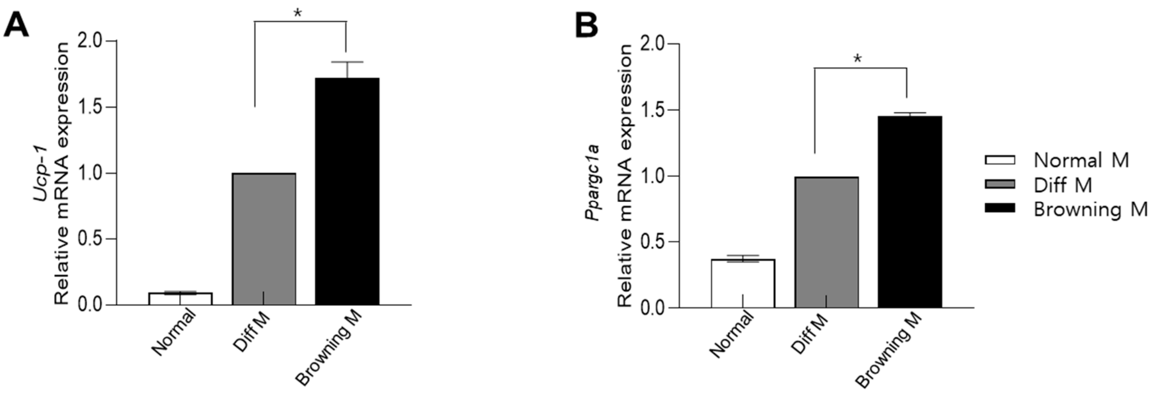

Supplementary Figure S2. Thermogenesis gene expressions at the differentiation and browning medium-treated 3T3-L1 adipocytes. 3T3-L1 preadipocytes were incubated with normal medium and differentiation medium for 7 days and maintenance and browning medium for 2 and the subsequent 5 days as described in Materials and Methods. *Ucp1* (A) and *Ppargc1a* mRNA (B) expression was measured by real-time RT-PCR. Normal, normal medium treated group; Diff M, differentiated medium group; Browning M, browning medium-treated group.

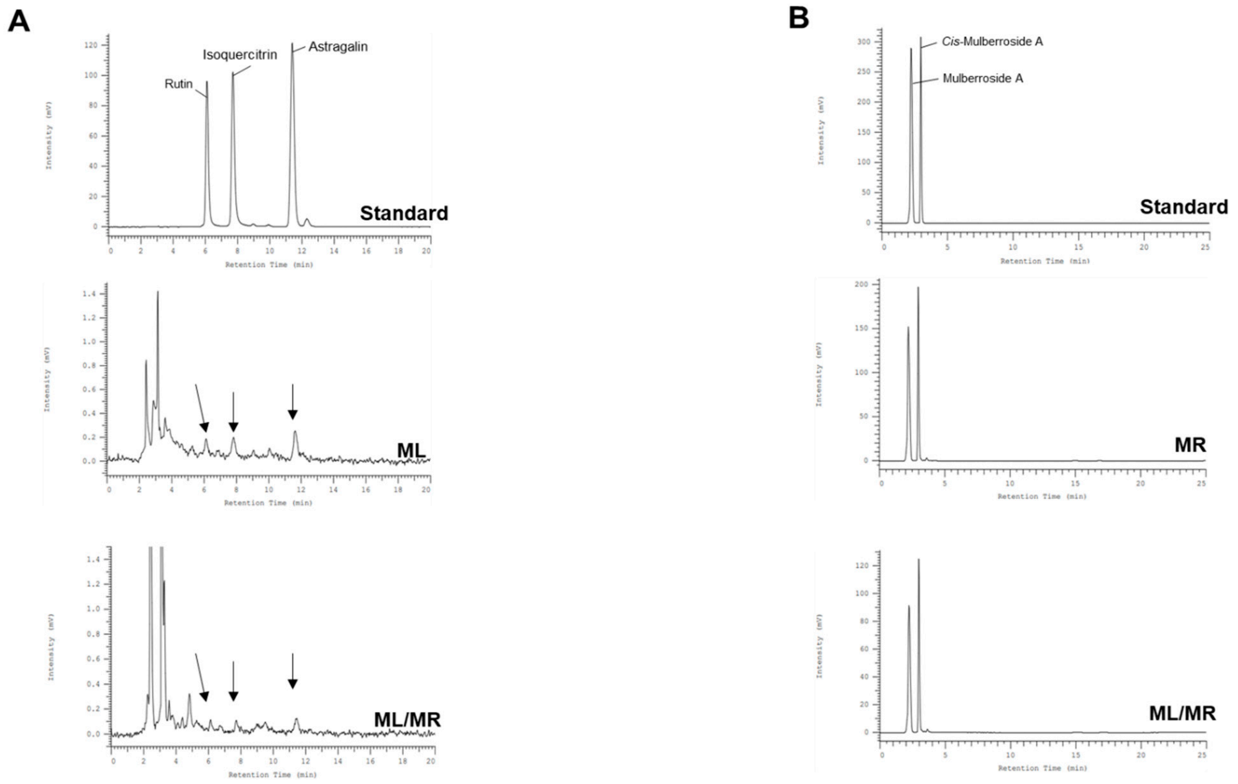

Supplementary Figure S3. Characteristic analysis of bioactive components in extracts of Mori Folium leaf and Mori Radicis Cortex roots by high-performance liquid chromatography. (A) Chromatogram at 370 and 320 nm showing retention time of rutin, isoquercitrin, and astraglin in ML, and ML/MR. and MR (B) Chromatogram showing retention time of mulaberroside A and *cis*-mulberroside A in ML, and ML/MR. ML; Mori Folium extract, MR; Mori Cortex Radicis extract, MF/MR; 1:1 mixture of MF/MR.

Supplementary Table S1. HPLC condition for analysis of standards

HPLC condition	Condition 1[1)]			Condition 2[2)]		
Column	Zorbax Eclipse Plus C18 column (250 mm × 4.6 mm, 5 μm)			Zorbax Eclipse Plus C18 column (250 mm × 4.6 mm, 5 μm)		
Column temp.	40 °C			40 °C		
Flow rate	1 mL/min			1 mL/min		
Wave length	370 nm			320 nm		
Injection volume	10 μL			10 μL		
Mobile solvent	A: Acetonitrile<br>B: Water			A: Acetonitrile<br>B: Water		
Mobile phase	Time (min)	A (%)	B (%)	Time (min)	A (%)	B (%)
	0	20	80	0	5	95
	20	20	80	25	30	70

[1)] Condition 1: Rutin, isoquercitrin, astragalin
[2)] Condition 2: Mulberroside A, *Cis*-mulberroside A

Supplementary Table S2. Real time PCR primer sequences

Gene	Forward (5′→3′)	Reverse (5′→3′)
UCP-1	GGCCTCTACGACTCAGTCCA	TAAGCCGGCTGAGATCTTGT
PGC-1α	GAAAGGGCCAAACAGAGAGA	GTAAATCACACGGCGCTCTT
Cited	GCGGTAAAAGATCGCAAGGC	TTGTAGAAGGGGTGGCAGTA
PRDM16	GATGGGAGATGCTGACGGAT	TGATCTGACACATGGCGAGG
Tbx1	CGAATGTTCCCCACGTTCCA	GTCTACTCGGCCAGGTGTAG
Fgf21	CGTCTGCCTCAGAAGGACTC	TCTACCATGCTCAGGGGGTC
β-actin	AAGACCTCTATGCCAACACAGT	AGCCAGAGCAGTAATCTCCTTC

Supplementary Table S3. Calibration standards for bioactive compounds

Compound	Calibration curve	Correlation coefficient (R^2)	Linear range (mg/L)	LOD (mg/L)	LOQ (mg/L)
Rutin	y =10373x + 1226.6	0.9994	2.5 ~ 100	3.05	9.26
Isoquercitrin	y = 12922x + 1216.8	0.9995	2.5 ~ 100	2.69	8.15
Astragalin	y = 18483x + 2689.5	0.9995	2.5 ~ 100	2.88	8.72
Mulberroside A	y = 13423x – 5381.2	0.9996	10 ~ 200	5.23	15.85
Cis-Mulberroside A	y = 7119.9x – 1468.5	0.9993	10 ~ 200	6.57	19.91
